# Supplementary material for: The Construction and Exploration of a Comprehensive MicroRNA Centered Regulatory Network in Foxtail Millet (Setaria italica L.)
Source: Front Plant Sci. 2022 May 6;13:848474. doi: 10.3389/fpls.2022.848474 (PMC9121102; doi:10.3389/fpls.2022.848474)
Supplement: Supplementary file 5 [file Data_Sheet_5.zip › Sit-miR399b_Seita.3G150400.1_984_TPlot.pdf]

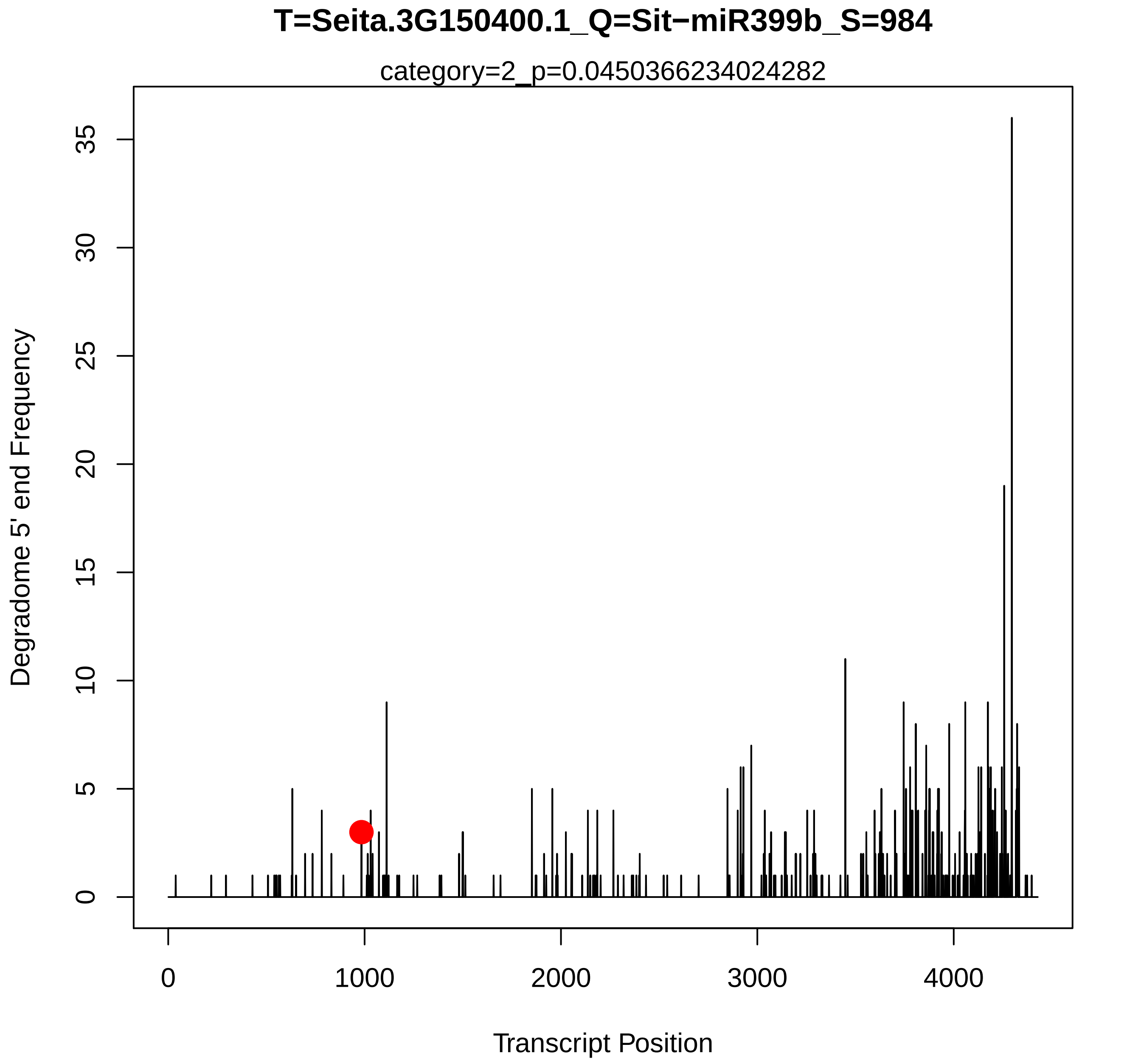

T=Seita.3G150400.1_Q=Sit−miR399b_S=984
category=2_p=0.0450366234024282
Degradome 5' end Frequency
0
5
10
15
20
25
30
35
0
1000
2000
3000
4000
Transcript Position
